# Energy expenditure estimation during activities of daily living in middle-aged and older adults using an accelerometer integrated into a hearing aid

**DOI:** 10.3389/fdgth.2024.1400535

**Published:** 2024-06-17

**Authors:** Jan Stutz, Philipp A. Eichenberger, Nina Stumpf, Samuel E. J. Knobel, Nicholas C. Herbert, Isabel Hirzel, Sacha Huber, Chiara Oetiker, Emily Urry, Olivier Lambercy, Christina M. Spengler

**Affiliations:** ^1^ Exercise Physiology Lab, Department of Health Sciences and Technology, ETH Zurich, Zurich, Switzerland; ^2^ Research & Development, Sonova AG, Stäfa, Switzerland; ^3^ Rehabilitation Engineering Laboratory, Department of Health Sciences and Technology, ETH Zurich, Zurich, Switzerland; ^4^ Zurich Center for Integrative Human Physiology (ZIHP), University of Zurich, Zurich, Switzerland

**Keywords:** metabolism, elderly, calories, earable, Fitbit, Garmin, Audéo

## Abstract

**Background:**

Accelerometers were traditionally worn on the hip to estimate energy expenditure (EE) during physical activity but are increasingly replaced by products worn on the wrist to enhance wear compliance, despite potential compromises in EE estimation accuracy. In the older population, where the prevalence of hearing loss is higher, a new, integrated option may arise. Thus, this study aimed to investigate the accuracy and precision of EE estimates using an accelerometer integrated into a hearing aid and compare its performance with sensors simultaneously worn on the wrist and hip.

**Methods:**

Sixty middle-aged to older adults (average age 64.0 ± 8.0 years, 48% female) participated. They performed a 20-min resting energy expenditure measurement (after overnight fast) followed by a standardized breakfast and 13 different activities of daily living, 12 of them were individually selected from a set of 35 activities, ranging from sedentary and low intensity to more dynamic and physically demanding activities. Using indirect calorimetry as a reference for the metabolic equivalent of task (MET), we compared the EE estimations made using a hearing aid integrated device (Audéo) against those of a research device worn on the hip (ZurichMove) and consumer devices positioned on the wrist (Garmin and Fitbit). Class-estimated and class-known models were used to evaluate the accuracy and precision of EE estimates via Bland-Altman analyses.

**Results:**

The findings reveal a mean bias and 95% limit of agreement for Audéo (class-estimated model) of −0.23 ± 3.33 METs, indicating a slight advantage over wrist-worn consumer devices (Garmin: −0.64 ± 3.53 METs and Fitbit: −0.67 ± 3.40 METs). Class-know models reveal a comparable performance between Audéo (−0.21 ± 2.51 METs) and ZurichMove (−0.13 ± 2.49 METs). Sub-analyses show substantial variability in accuracy for different activities and good accuracy when activities are averaged over a typical day's usage of 10 h (+61 ± 302 kcal).

**Discussion:**

This study shows the potential of hearing aid-integrated accelerometers in accurately estimating EE across a wide range of activities in the target demographic, while also highlighting the necessity for ongoing optimization efforts considering precision limitations observed across both consumer and research devices.

## 1 Introduction

Engaging in regular physical activity (PA)—defined as any bodily movement produced by skeletal muscles that results in energy expenditure (EE) (1)—is associated with a lower risk for numerous chronic diseases and premature death (2). Meeting PA guidelines is sufficient to elicit health benefits, in particular in previously sedentary people. These benefits appear to increase in a dose-dependent manner (2). However, about one-quarter of the Swiss population does not meet the requirements of at least 150 min of moderate-intensity PA or 75 min of high-intensity PA per week. This proportion increases to about one-third in individuals aged 75 years or older (3). In light of strong evidence linking PA to healthy aging (4), the precise monitoring and promotion of PA among middle-aged and older adults emerge as critical strategies for personalized prevention and enhancing public health.

The use of wearable devices, which measure acceleration in either a uni- or triaxial plane, represents a promising method for such monitoring. They are easy to use, unobtrusive, and have already been shown to estimate activity and/or EE with reasonably good accuracy in healthy adult populations (5, 6) as well as in patients with chronic disease (7–10). In addition, in a recent systematic review and meta-analysis, activity trackers have been shown as effective in promoting an increase in PA and reducing sedentary time in older adults (11). While this underscores the potential of these devices to estimate EE and promote PA in the middle-aged and older population, there is still an ongoing debate regarding the most appropriate sensor location.

Originally, accelerometers were worn on the hip, but they are now increasingly worn on the wrist. For example, the National Health and Nutrition Examination Survey initially relied on sensors worn on the hip to capture PA and sedentary behavior (12) and later switched to wrist-worn accelerometers (13). The main arguments typically raised in favor of wrist placement are continuous wearability, enabling sleep monitoring, and improved wear compliance (14). This was demonstrated by Huberty et al. (15), who reported that 24 h monitoring over seven consecutive days in middle-aged women is significantly more effective with wrist-worn sensors, achieving seven valid days of data for 95% of participants, compared to just 62% with hip-worn sensors. However, wrist-worn accelerometers generally provide less accurate EE estimates than hip-worn sensors (16–18). This supports the consensus that sensors positioned closer to the body's center of mass are more precise than those located more distally, such as on the arm or wrist. Although wrist sensors can accurately estimate EE during locomotion activities (19), disparities might emerge during activities with restricted arm movement (e.g., walking with a stroller) or during activities of daily living (ADL) involving a lot of arm movement (e.g., playing cards). Given that time spent in ADL increases with age (20), this disparity may become more pronounced in older populations. Indeed, Guediri et al. (21) found a greater discrepancy in EE estimates between hip and wrist-worn sensors in older compared to younger subjects under free-living conditions. This highlights the need for novel approaches to accurately capture EE in middle-aged and older populations, addressing the limitations of current methods, including the lower accuracy of wrist-worn sensors and the lower compliance associated with hip-worn sensors.

A promising strategy might involve the use of an accelerometer within a device that users already wear for extended periods throughout the day and that is situated sufficiently close to the body's center of mass to reflect the wearer's movements. Hearing aids might present such a viable option. With an average usage of 10 h per day (22), these devices are commonly worn by an increasing portion of the middle-aged and older population, a group particularly vulnerable to hearing loss. For instance, among US adults aged between 45 and 64, about 3% of men and 2% of women use hearing aids. For those aged 65 and older, the rate rises to 14% (23). This proportion would increase even further if more adults affected by hearing loss would use hearing aids. In a nationally representative sample of older US adults, 65% of adults aged 71 years and older had at least a mild hearing loss, but only 29% of them used hearing aids (24). In addition, hearing loss is associated with less PA in adults aged 60–69 years (25). Thus, monitoring and promoting PA within this population can yield significant health benefits.

Previous studies evaluating accelerometers worn around the ear (26–28) have shown promise in estimating EE but were limited to younger adults and a narrow range of activities, and none incorporated the accelerometer directly into a hearing aid. Addressing this gap and given the rising trend of wearables with health sensors (29), the current study aimed to explore whether a similar approach can effectively predict EE in a broader spectrum of ADL for middle-aged and older adults, specifically through an accelerometer integrated into a hearing aid. Thus, this research focused on adults aged 45–64 years, and those 65 years and older, comparing the accuracy of this new sensor placement with a research device worn at the hip and wrist, as well as wrist-worn consumer devices. We followed the features of phase I in the framework of Keadle et al. (30) and also included activities pertinent to an older population. We hypothesized that the EE prediction accuracy arising from a sensor worn at the ear would be comparable to a hip-worn sensor, yet better than wrist-worn devices.

## 2 Materials and methods

### 2.1 Participants

Sixty middle-aged and older adults (64.0 ± 8.0 years, BMI 24.4 ± 2.8 kg · m^−2^, 48% females) participated in this study (Table 1). Participants were recruited through word-of-mouth, advertisements on the university campus, and in local retirement homes. All 60 participants recruited also completed the study.

**Table 1:** Participant characteristics.

	Middle-aged (*N* = 30)	Older (*N* = 30)	*p*-value
Age [years]	57.2 ± 4.9	70.8 ± 3.6	<0.001
Sex (M/F)	13/17	18/12	0.196
Height [cm]	172 ± 8	171 ± 8	0.733
Weight [kg]	71.7 ± 11.8	72.8 ± 12.7	0.748
BMI [kg⋅m^−2^]	24.1 ± 2.5	24.7 ± 3.1	0.463
IPAQ [MET⋅min^−1^⋅week^−1^]	2,955 ± 1,876	2,520 ± 1,751	0.357
Resting $\dot{V}$O_2_ [ml⋅min^−1^⋅kg^−1^]	3.23 ± 0.58	3.08 ± 0.61	0.326
Fat mass [kg]	20.0 ± 6.3	21.2 ± 6.5	0.485
Lean mass [kg]	49.3 ± 8.9	49.1 ± 9.2	0.469
BMD [T-score]	0.30 ± 1.1	−0.34 ± 1.1	0.038
Systolic BP [mmHg]	119 ± 11	130 ± 14	<0.001
Diastolic BP [mmHg]	82 ± 11	80 ± 7	0.206
PWV [m⋅s^−1^]	7.8 ± 2.0	9.8 ± 2.5	<0.001
Handiness (R/L)	26/4	27/3	1.000

Shown are means ± SD. BMI, body mass index; BMD, bone mineral density, BP, blood pressure, PWV, pulse wave velocity; IPAQ, international physical activity questionnaire—short form; MET, metabolic equivalent of task; *p*-value, two-sided independent *t*-test for numerical variables, Pearson Chi-Square for Age, and Fischer's exact test for handiness.

To qualify for inclusion, subjects were required to be 45 years or older, in good health, and not taking any medication (except for some allowable health conditions and medications for participants aged 65 years and above—refer to exclusion criteria). Additional requirements included a BMI greater than 18.5 kg · m^−2^, the ability to perform all activities outlined in the study protocol, normal hearing or at max. mild hearing loss, and willingness to comply with the study's procedural guidelines (i.e., to refrain from intense exercise 48 h before testing; to abstain from any exercise 24 h before testing; to ensure a minimum of 7 h of sleep on the two nights before testing; to avoid alcohol on the evening before testing, as well as on the day of testing; to not consume any caffeinated food or beverages before testing on the day of the experimental visit; and to arrive fasted after at least 10 h without food intake for visit 2).

Subjects were excluded if they had a history of heart, cardiovascular, metabolic, or neurological disease (incl. seizures and cognitive impairment), a biomechanical dysfunction affecting the ability to perform all activities, ear canal pathologies, moderate or severe hearing loss, an existing implanted medical device that may interfere with data collection, skin allergies or sensitivity to materials or devices used in the experiments, and—specifically for the participants aged 65 years and above—medication influencing heart rate or EE and neurological, orthopedic, rheumatologic, or metabolic disorders influencing upper or lower limb function.

We aimed to recruit 30 adults aged between 45 and 64 years and 30 participants aged 65 years or older, with each group containing a BMI distribution encompassing normal weight, overweight, and obese categories and males and females in equal numbers (≥40% for each sex). The study was approved by the local ethics committee of ETH Zurich (EK 2022-N-44). Every participant gave written informed consent in accordance with the Declaration of Helsinki before participating in the experiment.

### 2.2 Protocol

#### 2.2.1 Overview

The study involved two separate visits to the Exercise Physiology Lab at ETH Zurich. The first visit, lasting approximately 2 h, aimed to verify the inclusion and exclusion criteria, and to familiarize the participants with the measuring devices and study procedures. The second visit, lasting between 6 and 8 h, constituted the experimental phase of the study and occurred no sooner than 48 h after the initial visit. Data collection took place between September 2022 and August 2023.

#### 2.2.2 Visit 1

##### 2.2.2.1 Informed consent and questionnaires

Upon arrival at the laboratory, study details were explained to the participants, followed by the collection of their informed consent. Demographic information, dietary habits, and lifestyle details were then assessed through three distinct questionnaires: A health screening questionnaire to assess cardiovascular and respiratory system health/risk, the International Physical Activity Questionnaire—Short Form (31), and a Daily Questionnaire to monitor participants' intake of caffeine, food, and medication, as well as their sleeping patterns and sporting activities over the past 2 days. Participants were then introduced to the study's equipment and procedures. This involved fitting them with the sensors and the facemask that is part of the portable ergospirometric system. Finally, the maximum achievable walking speeds (on both a flat surface and with a 10% incline) and running speeds (on a flat surface) on a treadmill were established for each participant.

#### 2.2.3 Visit 2

**Figure 1:**
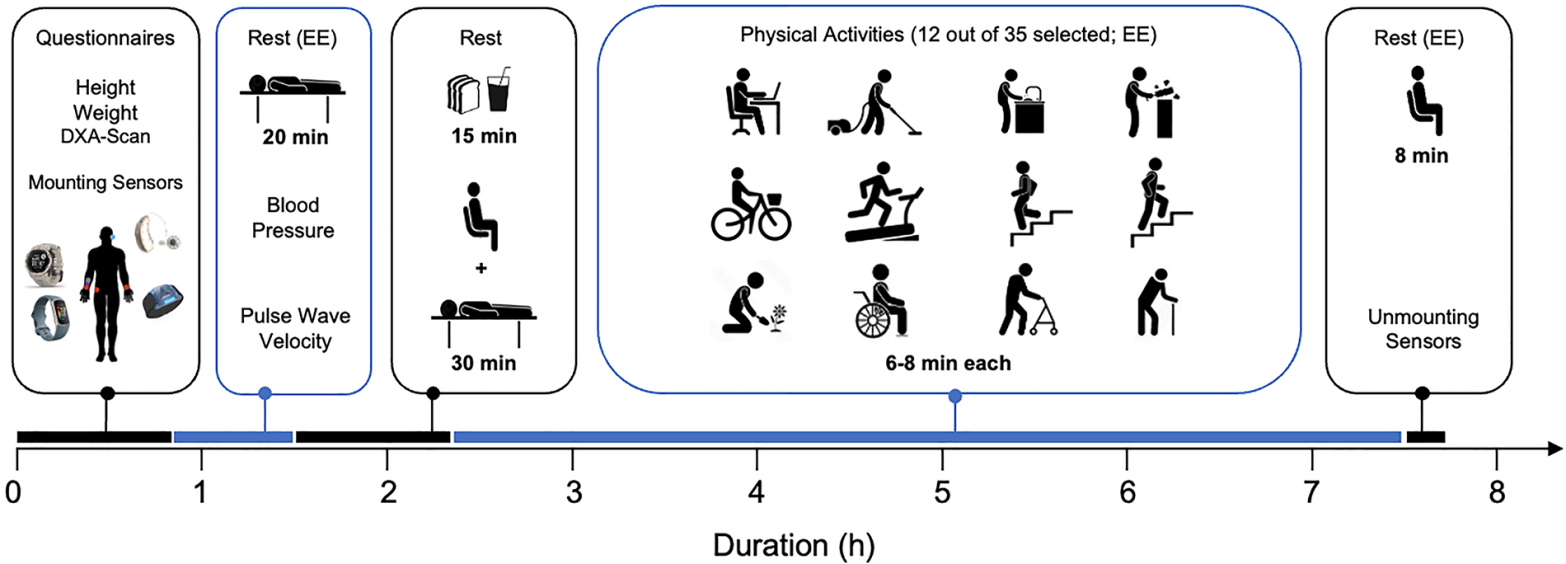
Overview of the experimental visit (visit 2). See text for details. EE, energy expenditure measurement.

Figure 1 illustrates an overview of visit 2. All activities, numbered 01–36 (refer to Table 2), were video recorded using a smartphone and subsequently downloaded onto a hard disk. To ensure optimal data quality, participants were instructed to refrain from speaking during all activities.

**Table 2:** Overview of physical activities.

Category	Nr.	Subgroup	Activity	Duration [min]	Activity description	*N*
Resting	01	A	Lie_bed_REE_20	20	Lying in bed on the back (on a pillow, before breakfast, fasted, 20 min)	60
	36	T	Sit_chair_post	8	Sitting on a chair after last activity	59
Sedentary and lying	02	B	Watch_TV	8	Watching TV (while sitting on a couch)	15
	03	B	Read_book	8	Reading a book (sitting on a chair)	15
	04	B	Crossword	8	Doing crossword puzzles	15
	05	B	Play_iPad	8	Playing on an iPad (Angry Birds)	15
	06	C	Write_paper	8	Writing on a piece of paper (copying from a paper document)	20
	07	C	Play_cards	8	Playing cards (Uno)	20
	08	C	Write_PC	8	Computer work (typing from a paper document)	20
Low intensity activities of daily living	09	D	Wash_dish	8	Dishwashing, 20 s washing plate, 20 s drying plate, repeat 12 times	45
	10	D	Prep_food	8	Food preparation (fruit salad)	15
	11	E	Clean_vacuum	8	Vacuum cleaning, 3 × 3 m square, 1 min per half square	33
	12	E	Clean_mop	8	Sweeping (mop) floor	27
	13	F	Dust_surface	8	Dusting surfaces (on different heights, vertical and horizontal)	15
	14	F	Hang_laundry	8	Hanging up laundry, 2 min per line. Laundry is removed by investigator	45
Activities with changing intensity or without physical displacement	15	G	Squats	8	Doing squats	12
	16	G	Gardening	8	Gardening (transplant plants, fetch plants, fetch water, and water)	34
	17	G	Stretch_yoga	8	Stretching/back exercises/gentle yoga	14
	18	H	Cycle_ergo	6	Bicycle ergometer (3 stages: 1 W · kg^−1^, 1.25 W · kg^−1^, 1.5 W · kg^−1^ each for 2 min)	60
Indoor activities related to locomotion	19	K	Walk_tm_flat_50	8	Walking on the treadmill without inclination at 50% of max. walking speed	24
	20	K	Walk_tm_flat_75	8	Walking on the treadmill without inclination at 75% of max. walking speed	24
	21	L	Walk_tm_inc10_50	8	Walking on the treadmill with inclination of 10% at 50% of max. walking speed	26
	22	L	Walk_tm_inc10_75	8	Walking on the treadmill with inclination of 10% at 75% of max. walking speed	22
	23	M	Run_tm_flat_50	8	Running on the treadmill without inclination at 50% of max. running speed	23
	24	M	Run_tm_flat_75	8	Running on the treadmill without inclination at 75% of max. running speed	25
Outdoor activities	25	N	Walk_cob	8	Walking on cobble stone at preferred speed	11
	26	N	Run_road	8	Running on a track at preferred speed	1
	27	O	Walk_up	8	Walking uphill at preferred speed	12
	28	P	Walk_down	8	Walking downhill at preferred speed	12
	29	Q	Climb_stairs	indiv.	Climbing stairs (6 floors, two times, rest in between)	44
	30	Q	Descend_stairs	indiv.	Descending stairs (6 floors, two times, rest in between)	48
	31	R	Cycle_road	8	Cycling on a paved road at self-selected speed	7
	32	R	Cycle_cob	8	Cycling on cobble stone	7
Activities with aids	33	S	Walk_stick	8	Walking with walking stick back and forth at preferred speed	21
	34	S	Walk_stroller	8	Walking with stroller back and forth at preferred speed	19
	35	S	Wheel_chair	8	Self-driving in a wheelchair back and forth at preferred speed	20

Activities were performed sequentially from A to T, with Sit_chair_post (Nr. 36) being the exception (performed at the end of the experimental visit). Participants performed one activity per subgroup letter (e.g., letter K: either Walk_tm_flat_50 or Walk_tm_flat_75). *N*, number of times an activity was performed.

##### 2.2.3.1 Daily questionnaire, anthropometrics, and sensor mounting

**Figure 2:**
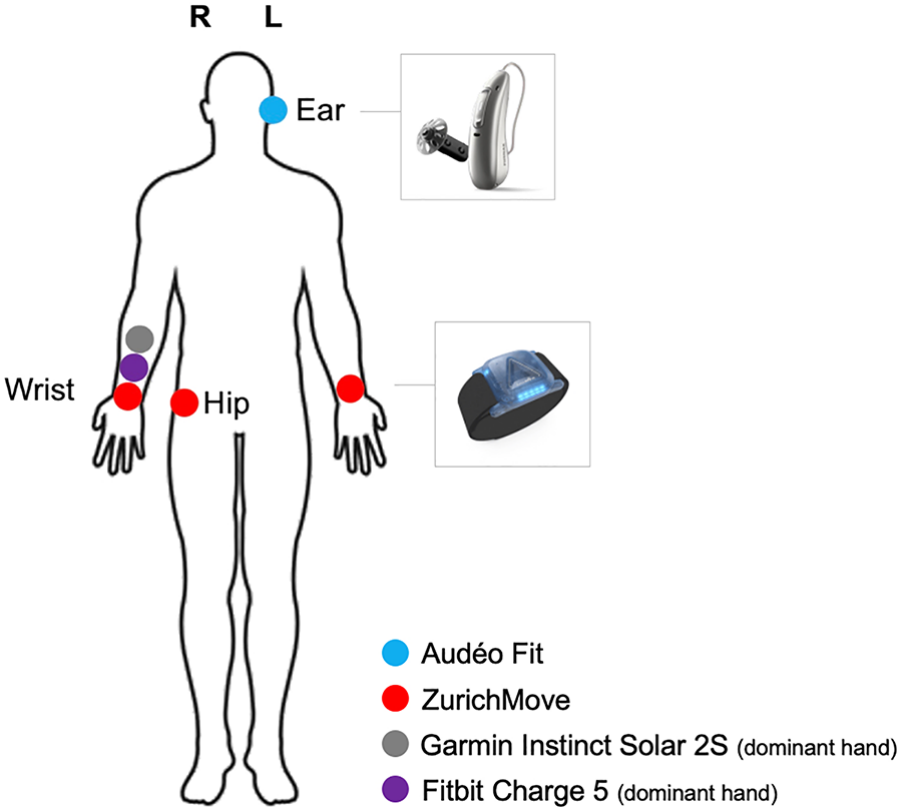
Overview of measurement devices and their location on the body.

Upon arrival at the laboratory, the participants' adherence to the study protocol was confirmed through the daily questionnaire. Then, measurements of weight, height, and body composition were taken as detailed in Section 2.3.3. The acceleration sensors were then positioned on the participants as detailed in Figure 2 and Section 2.3.1, ensuring they were securely fastened to avoid any displacement during the activities. Special care was taken to fit the facemask airtight on the face before starting the ergospirometric device. To ensure comfort of the participants, they were asked about their comfort in each break, and—in case of need—a respective strap was loosened during the break or the face mask (ergospirometric device) was removed and/or slight adjustments were made to the strapping, making sure not to change the position of any sensor.

##### 2.2.3.2 Resting energy expenditure, blood pressure, and pulse wave velocity

After completing the preparatory phase, the measurement of resting energy expenditure (REE) was conducted for 20 min (activity 01, see Table 2). During this time, participants were positioned supine on a stretcher. They were instructed to remain still and quiet. After the REE measurement, still lying supine on the stretcher, blood pressure was measured on the left upper arm, as detailed in Section 2.3.4. Immediately after this, pulse wave velocity was assessed, following the procedure described in Section 2.3.5.

##### 2.2.3.3 Breakfast and resting period

Participants then proceeded to a table where a standardized breakfast was provided. Subjects weighing less than 90 kg received a meal comprising 70 g of bread, 10 g of butter, 28 g of marmalade, 250 ml of orange juice, and water *ad libitum*, totaling 450 kcal. For those weighing 90 kg or more, the breakfast composition was identical, except for an increased bread portion of 105 g, totaling 550 kcal. All subjects were instructed to consume the entire meal at their preferred pace. After breakfast, participants rested for 30 min to minimize the impact of diet-induced thermogenesis on subsequent activities.

##### 2.2.3.4 Physical activities

Next, each participant engaged in 13 different activities, 12 of them chosen from a pool of 35 activities (Table 2); the 13th activity was performed by everyone as the final task (activity 36). During all activities, energy expenditure (ergospirometric device) and acceleration signals were continuously recorded (see Section 2.3). Activities were categorized into six categories: sedentary & lying, low-intensity activities, activities with varying intensity or not involving physical movement, indoor locomotion-related activities, outdoor activities, and activities requiring aids. For data processing and analyses, the activities were classified as detailed in Section 2.4.4. To ensure a balanced distribution, activities were pseudo-randomly allocated to participants. Each activity had a duration of 8 min, with two exceptions: cycling on the ergometer, which was performed for 6 min, and stair climbing and descending, taking approximately 1.5 min, varying with the participant's chosen walking speed. Before each activity, there was a 2 min sitting period on a chair. In between activities, a standardized break of at least 4 min was provided. After the last activity, all devices were removed, and data was downloaded locally on a computer.

### 2.3 Measurement devices

#### 2.3.1 Activity monitors

Acceleration in the three-dimensional plane was measured using a commercially available hearing aid (Figure 2) with integrated 3-axial accelerometers (Audéo Fit, Sonova AG, Stäfa, Switzerland, size 30.6 × 12.3 × 8.1 mm, weight 2.3 g, sampling frequency 200 Hz), two commercially available activity trackers (Fitbit Charge 5, Fitbit Inc., San Francisco, USA and Garmin Instinct Solar 2S, Garmin Ltd., Olathe, Kansas, USA), and a ten-axis inertial measurement unit used in research (ZurichMove, Rehabilitation Engineering Lab, Zurich, Switzerland, size 46 mm × 35 mm × 13 mm, weight 18 g, measurement range ± 16 g, resolution 1/2,048 g, sampling frequency 50 Hz). The sensors were positioned at the wrists, right hip, and left ear, as illustrated in Figure 2.

Garmin and Fitbit were attached to the wrist of the dominant arm, while ZurichMove sensors were placed on the hip and both wrists. The hip sensor was fixed via an elastic band with a silicon inlay around the body on hip level, placed directly on the skin. This way, the sensor located on the level of the iliac crest on the lateral edge.

Successful data transmission during the test was ensured either by visual inspection of the real-time data feed (ergospirometric device, Audéo, Garmin, Fitbit) or by visually checking the status indicator on ZurichMove. Audéo data was transferred and stored in real-time to a smartphone app during the activities. Garmin and Fitbit both recorded on the device and data was transferred to a smartphone app at the end of all experimental recordings of the day. ZurichMove recorded on the sensor itself and the data was transferred to a laptop via a docking station at the end of all experimental recordings of the day. Data from the ergospirometric device was transferred in real-time to a laptop with a dedicated software.

#### 2.3.2 Indirect calorimetry

A portable, battery-operated, ergospirometric device (Oxycon Mobile, Vyair Medical, Höchberg, Germany [53 participants] or Metamax 3B, Cortex, Leipzig, Germany [7 participants]) served as a basis for the calculation of reference EE. It recorded, on a breath-by-breath basis, parameters related to ventilation and gas exchange (O_2_ uptake, $\dot{V}$O_2_; CO_2_ output, $\dot{V}$CO_2_), transmitting the data telemetrically to a computer. For technical reasons, the Oxycon-system needed to be replaced shortly before the end of the 1-year duration of data acquisition period (percentage of participants using the Metamax-system was similar between the calibration and validation groups, see Section 2.4.3). Each system was mounted on a harness worn on the back. Before each test, a calibration was carried out according to the manufacturer's instructions, which consisted of (1) recording of the ambient conditions, (2) calibration of the flow sensor using a 3-liter calibration syringe and (3) calibration of the O_2_ and CO_2_ sensors using a gas cylinder with known gas concentrations (5% CO_2_, 16% O_2_).

#### 2.3.3 Body composition measurements

Height and weight measurements were taken using a stadiometer and an Omron BF511 digital scale (Omron, Kyoto, Japan). Segmental fat and lean body mass proportions, relative to the total body mass, were determined using a calibrated Lunar iDXA densitometer (GE Healthcare, Madison, WI, USA). The data were analyzed following the manufacturer's guidelines, with automatic processing conducted by the device's proprietary software.

#### 2.3.4 Blood pressure

Systolic and diastolic blood pressure was measured on the left upper arm using a cuff and an automated blood pressure monitor (Metronik BL-6, Metronik, Aue, Germany). Before the measurement, subjects were lying comfortably for at least 20 min (see REE measurement, Section 2.2.3). At least 3 valid measurements were taken with a 1 min break in between the measurements. The average of 3 valid measurements was taken as the final value.

#### 2.3.5 Pulse wave velocity

Pulse wave velocity was measured using two piezoelectric pressure sensors (placed manually on the carotid and femoral artery via palpation) sampling at 1kHz and attached to an acquisition unit (Complior, Alam Medical, Saint Quentin Fallavier, France). Before the measurement, subjects were lying comfortably for at least 20 min (see REE measurement, Section 2.2.3). At least 3 valid measurements were taken. The average of 3 valid measurements was taken as the final value.

### 2.4 Data processing and analysis

#### 2.4.1 Data preprocessing

All raw data were preprocessed using MATLAB R2023a (The MathWorks Inc., Natick, Massachusetts, USA). The primary objective of this preprocessing was to systematically reorganize the data for each participant, categorizing it by activities. Prior to each testing session, clocks of all devices were synchronized. Timestamps from the Audéo hearing aid served as the reference for timing of the activities, and the data from all other devices were adjusted to align with this time.

#### 2.4.2 Processing

The reference EE was calculated from $\dot{V}$O_2_ and $\dot{V}$CO_2_ values according to Weir (32) using the in-built formula of the metabolic device (Oxycon Mobile) and applied to both systems:

$$\mathrm{EE}\;(\mathrm{kcal}\cdot \mathrm{da}y^{-1})=1.59\times \dot{V}CO_{2}+5.68\times \dot{V}O_{2}-2.17\times \mathrm{UN}$$

with UN = 15 g · day^−1^. Outlier removal for ventilation and gas-exchange variables involved a two-step process: conservative removal of non-physiological values and deletion of values that deviated more than two standard deviations from the local 30 s mean.

In order to estimate EE from acceleration data, the calculation of “acceleration counts” (or simply “counts”) was required (see Section 2.4.4). These counts were obtained using a simplified version of the method developed by Actigraph (33). In order to capture signal components related to slow and fast movements (e.g., slow walking and running) while removing high-frequency noise (e.g., vibrations), the *x*, *y*, and z acceleration data for the entirety of the signal were bandpass filtered [lower cutoff frequency = 1 Hz, upper cutoff frequency = 12.5 Hz, based on (34)], the squared magnitude was computed as x^2^ + y^2^ + z^2^, and 1 min epochs were approximated by applying a first order low pass filter with 1 min time constant.

Ventilation and gas-exchange variables, and accelerometer counts were averaged over the last 4 min for all activities except climbing stairs, descending stairs, and the three stages of cycling on the ergometer, for which the last 20 s were used. This was done to ensure that participants reached a steady-state V̇O_2_. The reference metabolic equivalent of task (MET) for each participant and activity was calculated by dividing V̇O_2_ with resting V̇O_2_ derived from activity 01 (REE). For Fitbit and Garmin, METs were calculated by dividing EE (in kcal) by the subject's weight (in kg) and the duration of the activity (in h), assuming 1 MET = 1 kcal · kg^−1^ · h^−1^ (35). METs from Audéo and ZurichMove sensors were derived as described below (Section 2.4.4 EE estimation).

#### 2.4.3 Calibration and validation groups

Subjects were quasi-randomly split into a calibration and validation group using an inbuilt randomization function in MATLAB. Conditions were set to allocate 44 subjects to the calibration group (73%; 22 middle-aged and 22 older) and 16 to the validation group (27%; 8 middle-aged and 8 older). The calibration group served to develop the EE estimation models, while the validation group was used to run statistical analyses.

#### 2.4.4 EE estimation

To account for real-world constraints (embedded device with limited computational power, energy storage, and memory), a low-complexity approach was used for the model implementation. The EE per activity *p* was estimated by linearly mapping (36) the acceleration counts to MET as:

$$\mathrm{ME}T_{p}=a_{k}\;\times \;\mathrm{count}s_{p}\;+\;q_{k}$$

where the slope $a_{k}$ and intercept $q_{k}$ per activity class were determined via linear regression from the counts and reference MET (from ergospirometric device) of all participants in the calibration group. The performed activities were mapped to activity classes based on their expected similarities in their accelerometer signals and MET ranges. Activity classes were either estimated based on the accelerometer signal (class-estimated approach) or manually assigned to one of the following classes: *LaySit, Sedentary, ADL, Stationary, WalkFlat, WalkUp, WalkDown, Run*, or *Aid* (class-known approach). The class-estimated approach was used to determine the performance of the Audéo EE estimation model and to compare it to the internal EE estimates from Fitbit and Garmin. The class-known approach was used to compare Audéo with ZurichMove sensors. The EE for participant *i* during activity *p* was finally obtained as:

$$EE_{i,p}\;(\mathrm{kcal})=\mathrm{ME}T_{p}\;\;\times \;\mathrm{BM}R_{i}$$

where $\mathrm{BM}R_{i}$ is the participant's basal metabolic rate, according to the Müller equation (37):

$$\begin{array}{r} \mathrm{BMR}\;(\mathrm{kcal}\cdot d^{-1})=\;(0.047\times \mathrm{weight}+1.009\times \mathrm{sex}-0.01452\\ \;\times \;\mathrm{age}+3.21)\times 239\; \end{array}$$

with sex = 0 for females and 1 for males, weight in kg, age in years, and 239 being the conversion factor from MJ to kcal. The selection of the formula was based on its satisfactory performance in estimating BMR within our study sample (mean absolute bias = 13.3%).

In order to contextualize accuracy, rather than presenting it solely in terms of MET errors, an estimation of the EE over a standardized whole day of hearing aid use (10 h) (22) was calculated. First, based on reference METs, activities were classified into the four intensity levels “very light”, “light”, “moderate”, and “vigorous” according to the intensity criteria described by Garber et al. (38) for older adults aged ≥65 years. Note that the levels “vigorous” and “near maximal to maximal” were combined in order to be compatible with the estimates used for the proportion of time spent in different activity classes. Daily kcal counts for individual participants were then calculated for, and summed over, the four different intensity classes according to the Formula:

$$EE_{i}\;(\mathrm{kcal}\cdot 10\;h^{-1}{)}_{i}=\overset{4}{\underset{k=1}{\sum }}\mathrm{ME}T_{i,k}\;\times \;\mathrm{BM}R_{i}\;\times \;P_{k}\;\times \;0.416$$

Where EE_i_ represents the estimated total kcal participant *i* burned over 10 h, MET_i,k_ the median estimated MET of all activities within intensity class *k* for participant *i*, BMR_i_ the estimated BMR of participant *i*, 0.416 the proportion of 10 h relative to 24 h, and *P_k_* the proportion of hearing aid use time that older adults spend in intensity level *k*. These proportions were approximated as: 73% very low, 17% low, 9% moderate and 1% vigorous intensity based on data of 18,000 Sonova hearing aid users. These approximations appear broadly consistent with other literature (25).

#### 2.4.5 Statistical analysis

The performance of the EE estimation models in the validation group was analyzed using Bland-Altman plots (39) comparing estimated and reference METs and calculating mean bias, 95% limits of agreement (LoA), and mean absolute errors (MAE). Analyses were performed for all activities except eating breakfast and postprandial resting measurements. The class-estimated model was used to quantify the Audéo performance and to compare it to Fitbit and Garmin. The class-known model was used to compare Audéo with ZurichMove sensors. Two-sided independent *t*-tests were used to test whether mean biases differed significantly from zero.

Bland-Altman analyses were also performed separately for separate activities, intensity classes, and age groups in the validation group. For the total daily EE, a Bland-Altman analysis was performed over the whole dataset. To assess whether Audéo was able to accurately detect a within-subject change in intensity, the change in MET between flat walking (activities 19 and 20) and flat running (activities 23 and 24) was compared with the reference method using regression analysis.

Two-sided independent *t*-tests, Pearson Chi-Square and Fischer's exact test were used to compare demographic and anthropometric data between the two age groups and between the calibration and validation groups. All analyses were performed using MATLAB R2023a. Significance was set as *p* < 0.05.

## 3 Results

### 3.1 Participant characteristics

Thirty middle-aged participants of median [range] age 59 [46–64] years (57% females) and 30 older participants [71 (65–78) years; 40% females] completed the study (see Table 1). On average, older participants had lower bone mineral density and higher systolic blood pressure and pulse wave velocity compared to middle-aged participants. There were no significant differences between the calibration and validation groups for the variables tested (see Supplementary Table S1).

### 3.2 Energy cost of physical activities

**Figure 3:**
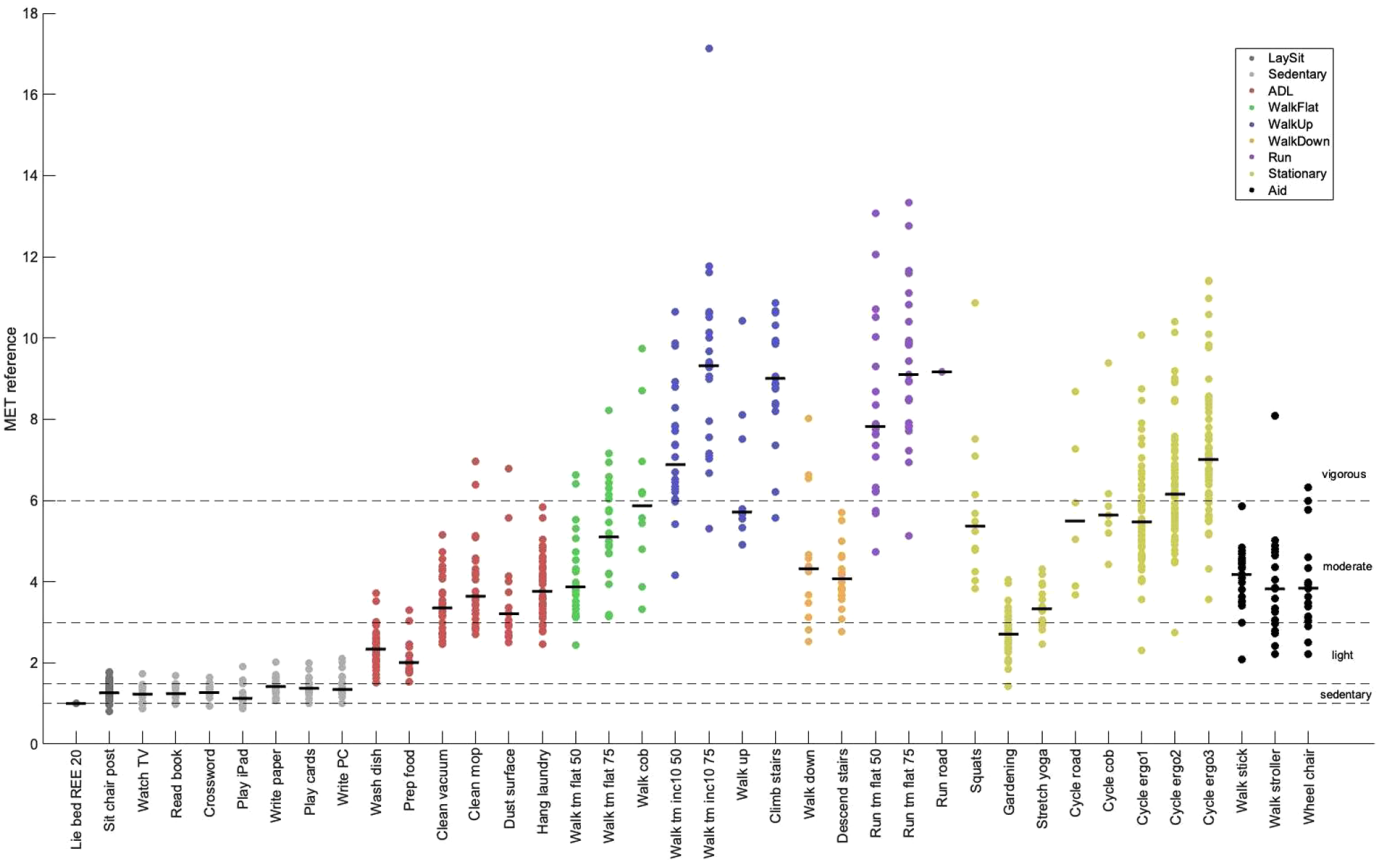
Metabolic equivalent of task (MET) by activity measured by the reference method. Shown are individual means (dots) and group medians (lines) for each activity. Refer to Table 2 for a description of the activities. The dotted lines show the thresholds for sedentary behavior (MET < 1.5), light intensity (1.5 ≤ MET < 3.0), moderate intensity (3.0 ≤ MET < 6.0), and vigorous-intensity physical activity (MET ≥ 6.0). ADL, activities of daily living; 50/75, 50%/75% of max. walking or running speed; inc10, 10% inclination.

Figure 3 shows the reference EE, as measured by the ergospirometric device, in METs for all activities. The median METs for most activities fell within the expected range (i.e., light intensity), except for some household activities, such as vacuum cleaning, cleaning with a mop, dust wiping, and hanging laundry, which were categorized into the moderate intensity category.

### 3.3 EE estimation

**Figure 4:**
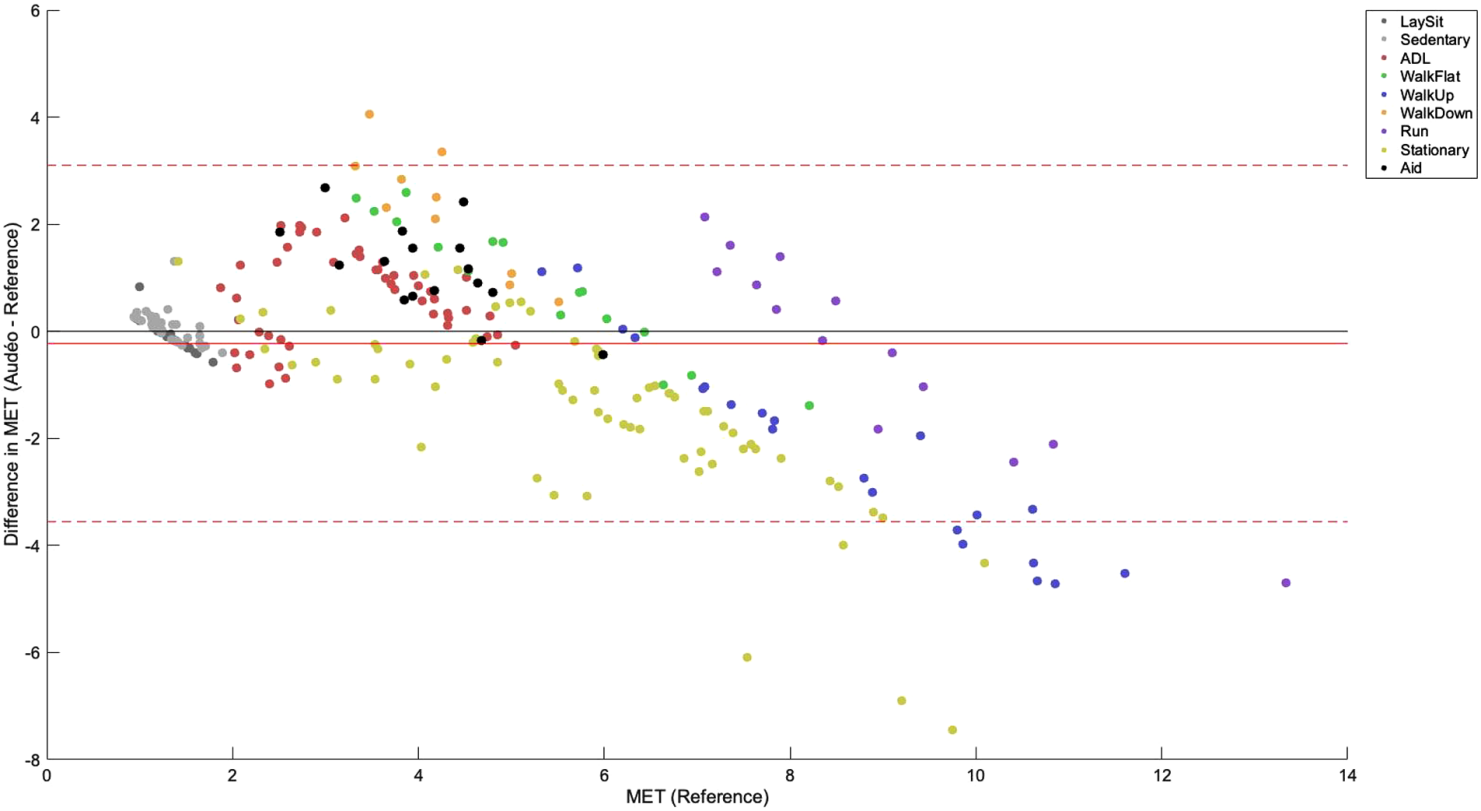
Bland-Altman plot for Audéo (class-estimated). The solid red line shows the mean bias, while the dotted lines indicate the upper and lower 95% limits of agreement. Colored dots show different activity classes. ADL, activities of daily living; MET, metabolic equivalent of task.

Mean bias for the Audéo class-estimated model was −0.23 METs (see Figure 4), differing significantly from zero (*p* = 0.031). Lower and upper LoA were −3.56 and 3.10 METs, respectively. MAE amounted to 1.19 METs.

**Figure 5:**
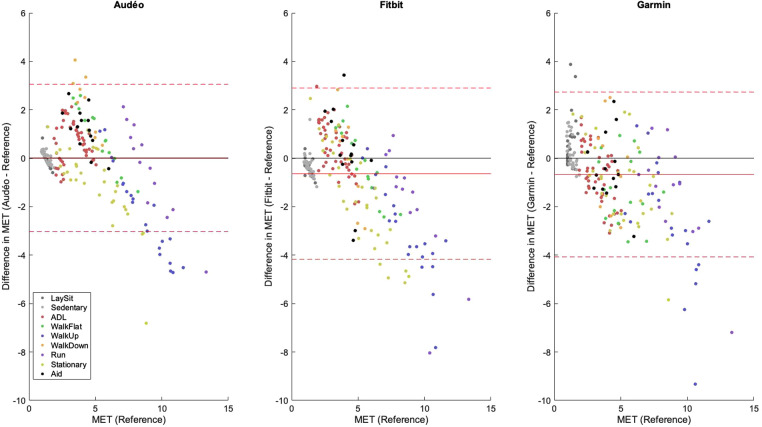
Bland-Altman plots for Audéo (class-estimated), Fitbit and Garmin. The solid red line shows the mean bias, while the dotted lines indicate the upper and lower 95% limits of agreement. Colored dots show different activity classes. ADL, activities of daily living; MET, metabolic equivalent of task.

The performance metrics for Audéo (class-estimated) vs. Fitbit vs. Garmin are shown in Table 3 and Figure 5. Note that for this comparison, two participants had to be excluded from the analysis as their Fitbit data could not be downloaded. To facilitate the comparison to the 6 min data from Fitbit and Garmin, the three stages of activity 18 (Cycle_ergo) were averaged into a single value.

**Table 3:** Performance metrics for Audéo (class-estimated), Fitbit, and Garmin.

	Mean bias [MET]	*p*-value	Lower LoA [MET]	Upper LoA [MET]	MAE [MET]
Audéo	0.02	0.879	−3.02	3.06	1.07
Fitbit	−0.64	<0.001	−4.17	2.90	1.29
Garmin	−0.67	<0.001	−4.07	2.74	1.32

MET, metabolic equivalent of task; LoA, limit of agreement; MAE, mean absolute error, *p*-value, two-sided independent *t*-test (mean bias different from zero).

Performance metrics for Audéo vs. ZurichMove (both class-known) are shown in Table 4. Note that with the approach used in this study, it was not possible to build an EE estimation model for the wrist sensors because of the missing linear relationship between counts and METs in the wrist data for the majority of the activity classes.

**Table 4:** Performance metrics for Audéo and ZurichMove (both class-known).

	Mean bias [MET]	*p*-value	Lower LoA [MET]	Upper LoA [MET]	MAE [MET]
Audéo	−0.21	0.008	−2.72	2.30	0.89
ZurichMove Hip	−0.13	0.101	−2.62	2.36	0.88

MET, metabolic equivalent of task; LoA, limit of agreement; MAE, mean absolute error; *p*-value, two-sided independent *t*-test (mean bias different from zero).

### 3.4 Sub-analyses

#### 3.4.1 Activities and intensities

**Figure 6:**
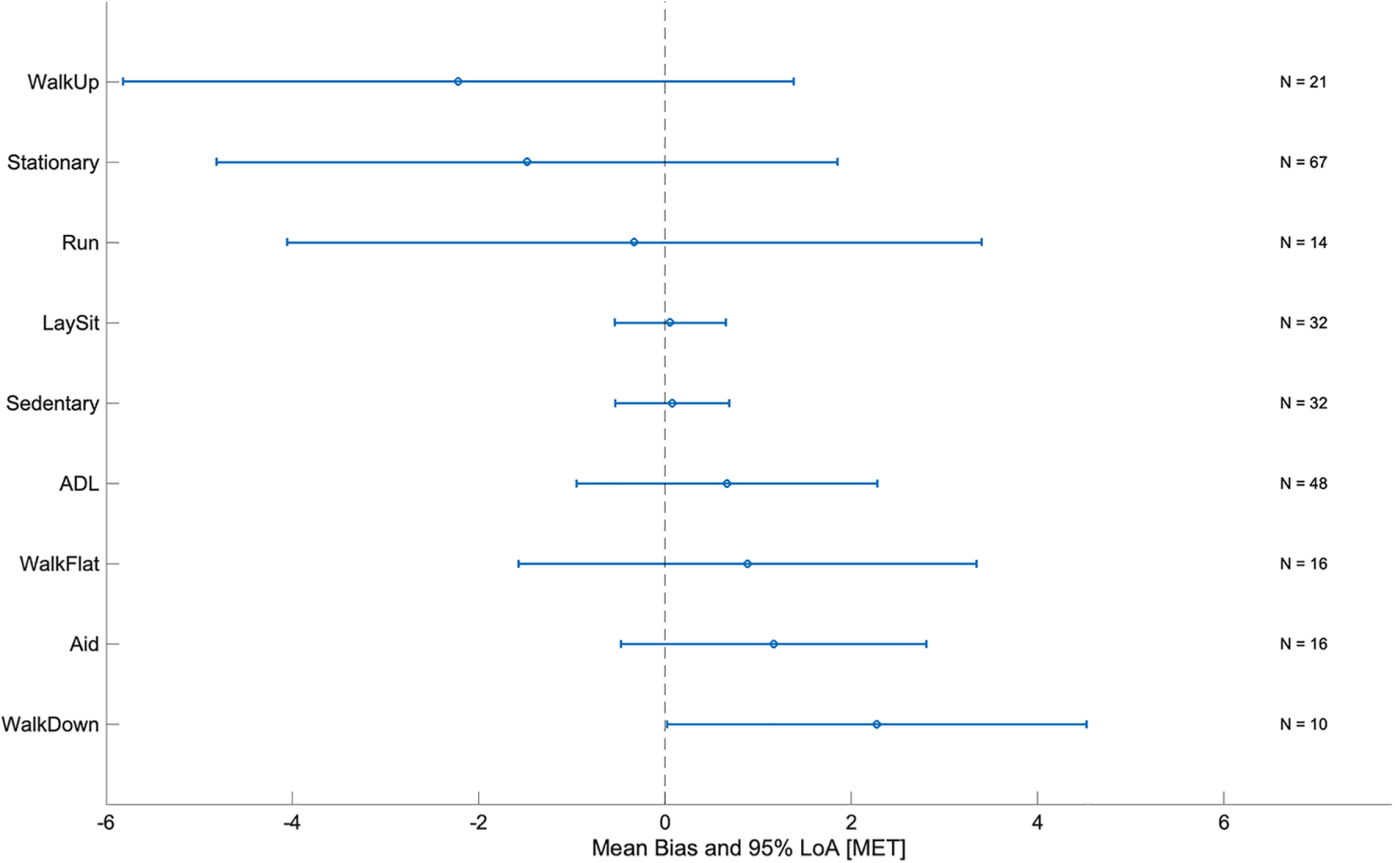
Mean bias and limits of agreement (LoA) by activity class for Audéo. ADL, activities of daily living; MET, metabolic equivalent of task; N, number of subjects (validation group).

Figure 6 shows the mean bias and LoA of the Audéo EE estimation for the different activity classes mentioned in Section 2.4.4. Mean bias was lowest for resting and sedentary activities and largest for activities performed on an incline (e.g., climbing and descending stairs or walking uphill). Mean bias and LoA increased with increasing intensity (see Table 5). For a detailed view discerning each single activity, refer to the online Supplementary Figure S1.

**Table 5:** Performance metrics by intensity level for the Audéo class-estimated model.

	Mean bias [MET]	Lower LoA [MET]	Upper LoA [MET]	MAE [MET]
Sedentary and light intensity	0.21	−1.13	156	0.46
Moderate intensity	0.62	−1.92	3.15	0.97
Vigorous intensity	−1.99	−5.65	1.68	1.39

MET, metabolic equivalent of task; LoA, limit of agreement; MAE, mean absolute error.

#### 3.4.2 Average error over a day

**Figure 7:**
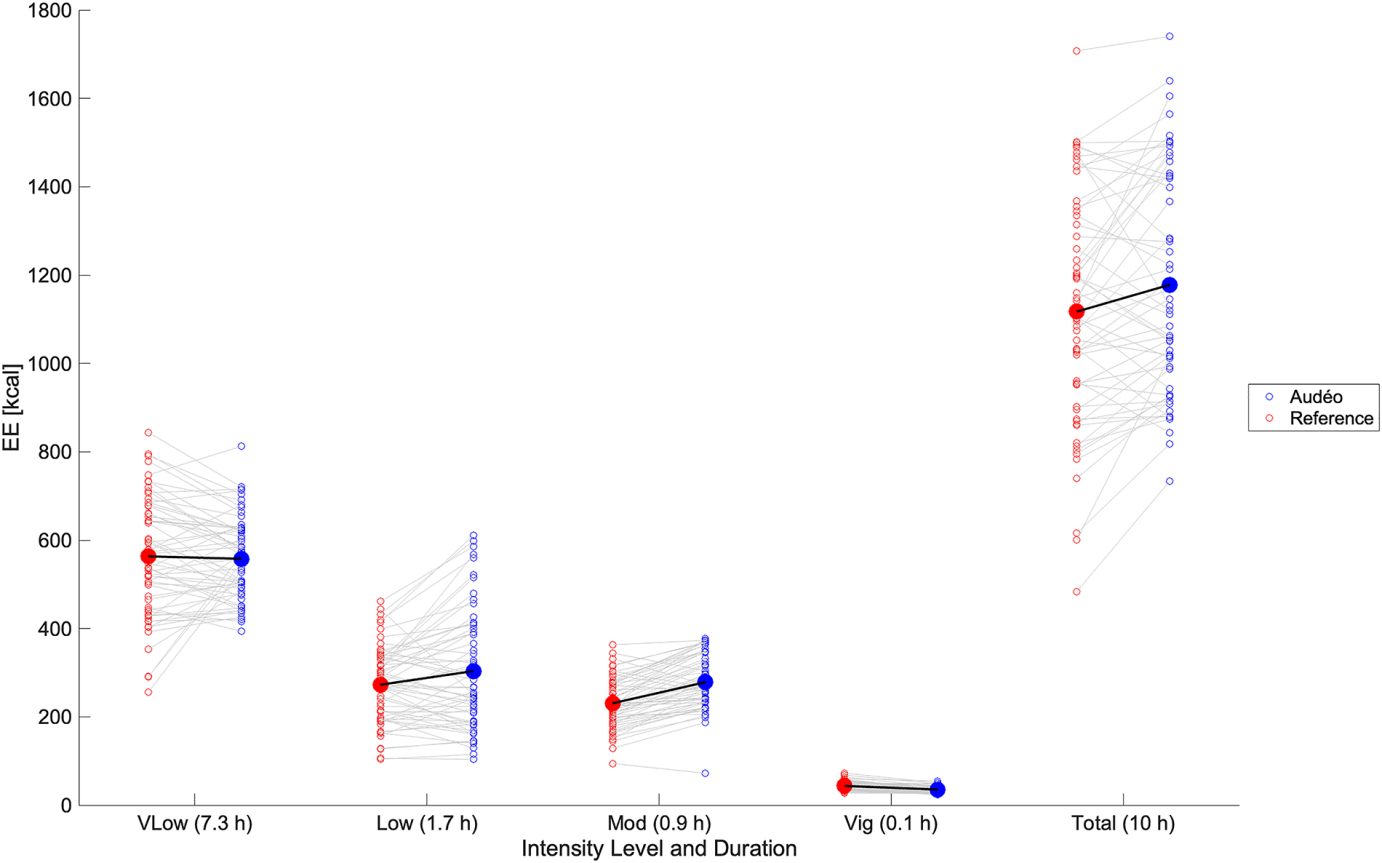
Daily caloric estimation: comparison between Audéo and reference. Empty circles show individual data, filled circles show mean values. Energy expenditure was summed over activities of very low intensity (vLow), low intensity (Low), moderate intensity (Mod), and vigorous intensity (Vig). EE, energy expenditure*.*

The total daily caloric estimation during wake-time (10 h) using reference EE values was 1,139 kcal. Mean bias for Audéo was +61 kcal (see Figure 7), with lower and upper LoA being −241 kcal and +363 kcal. MAE amounted to 131 kcal. Accuracy and precision for Fitbit (mean bias −111 kcal and lower and upper LoA −435 and +213 kcal, respectively) were comparable to Audéo. Compared to Audéo and Fitbit, Garmin showed similar accuracy but lower precision (mean bias 136 kcal and lower and upper LoA −716 and +989 kcal, respectively).

#### 3.4.3 Age subgroups

The EE estimation model performed slightly better in the older subgroup compared to the younger subgroup, as indicated by a lower mean bias (−0.13 METs in older vs. −0.33 METs in middle-aged participants), narrower LoA (−3.27 to +3.01 METs vs. −3.84 to +3.18 METs), and lower MAE (1.18 METs vs. 1.20 METs, *p* = 0.896).

#### 3.4.4 Within-subject change in intensity

**Figure 8:**
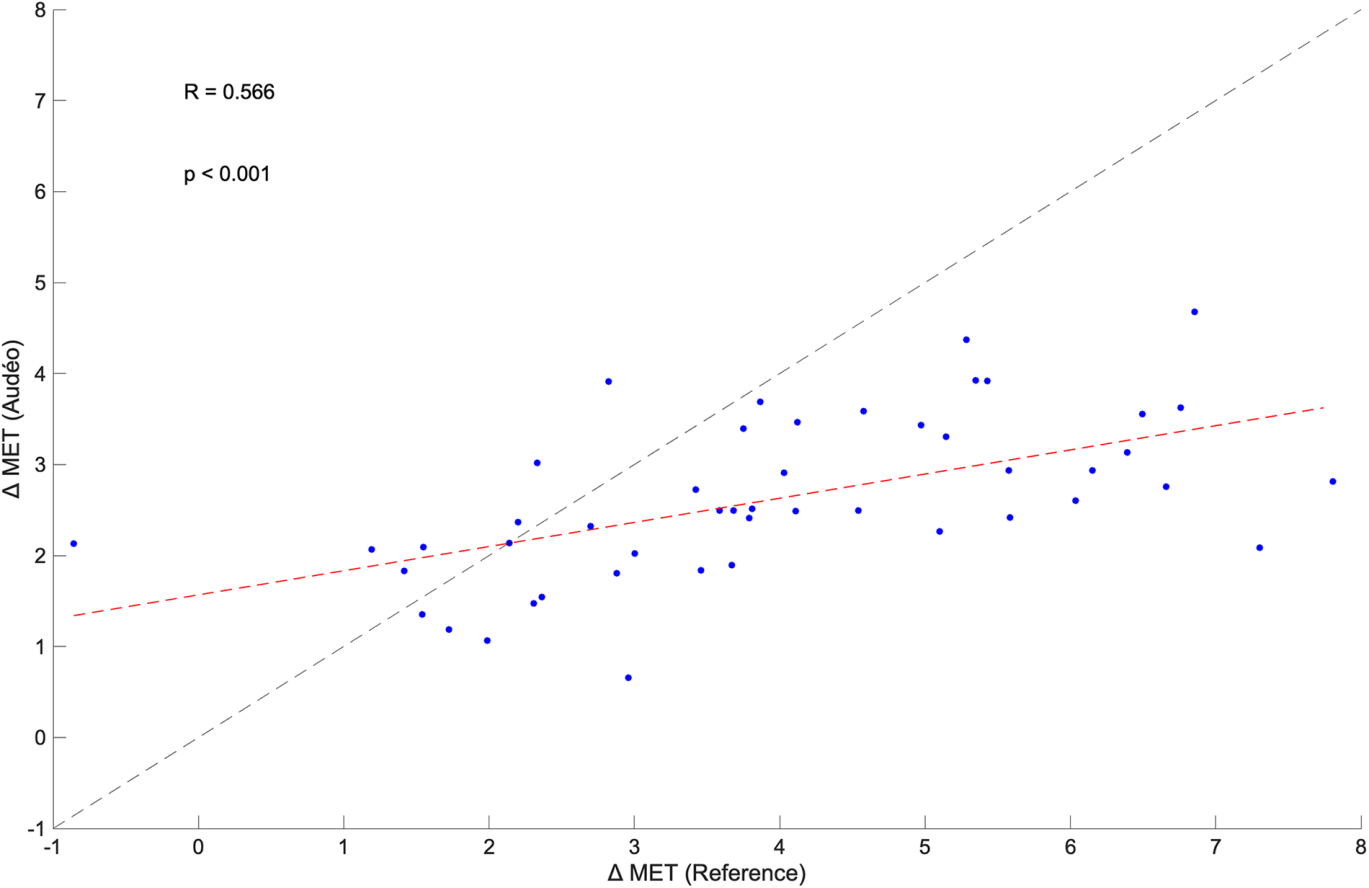
Within-subject correlation between the change in metabolic equivalent of task (MET) for Audéo and reference between walking and running. The dashed black line shows the identity line, while the dashed red line is the regression line.

The within-subject changes in METs from walking flat to running flat for Audéo and the reference were positively correlated (*R* = 0.566, *p* < 0.001) (see Figure 8). The mean change in METs for Audéo amounted to 2.63 METs, as opposed to the reference which had a mean change of 3.99 METs. Mean bias of change was −1.36 METs, with lower and upper LoA being −4.42 and 1.69 METs. MAE of change was 1.65 METs.

## 4 Discussion

This study aimed to predict EE in middle-aged and older participants in a broad spectrum of ADL (including activities with aids) using an accelerometer integrated into a hearing aid and comparing it at the same time to other research and consumer devices located on the wrist and hip. Bland-Altman analyses show good overall accuracy (low mean bias; Audéo vs. Reference: 0.23 METs) but low precision (wide LoAs; Audéo vs. Reference: ± 3.33 METs). Performance was slightly superior to wrist-worn consumer accelerometers and equivalent to a research accelerometer placed on the hip when the same modeling approach was used.

### 4.1 EE estimation

Across all activities, the Audéo prediction model (class-estimated) demonstrated a minor underestimation of 0.23 METs, along with a wide LoA of ± 3.33 METs. These findings align with other studies that used ear-level accelerometers to predict EE. For example, Atallah et al. (26) used an ear-worn inertia sensor during 11 ADLs (lying, standing, computer work, vacuuming, stairs, walking, running, cycling, and rowing) in 25 healthy young subjects. This resulted in an overall mean bias of 0.04 METs and a LoA of ± 3.65 METs. Similarly, Bouarfa et al. (27) developed an EE prediction model using 25 young subjects involving 10 ADLs (same as in Atallah et al., but without rowing). They reported a mean absolute deviation below 1.2 METs, i.e., identical to the MAE found in this study.

Good accuracy and low precision are typically also observed in other studies that use research or consumer devices to predict EE. For example, Crouter et al. (40) investigated the performance of the Actigraph and Actical devices (both worn on the waist), and the AMP-331 monitor (worn on the ankle) during 18 different leisure and sporting activities (e.g., lying, computer work, vacuuming, walking, running, stairs, basketball) to predict MET in 48 younger to middle-aged adults. The Bland-Altman plots show mean biases of about −0.5, −1.0, and −2.5 METs for Actigraph, Actical, and AMP-331, respectively, and LoAs of about ± 3.5, ± 3.0, and ± 4.0 METs. This data also aligns with our finding that sensors located closer to the body's center of mass (including the ear), tend to outperform devices positioned on the limbs when the goal is to predict EE in a wide range of ADL. Literature supports this observation, indicating that wrist-worn accelerometers generally yield less accurate EE estimates compared to those worn on the hip (16–18). In theory, this difference could be attributable to different device grades, as research devices are typically being worn on the hip or chest, while consumer devices are worn on the wrist. For example, Chowdhury et al. (41), comparing consumer monitors (Microsoft Band, Apple Watch, and Fitbit Charge HR) with a research device (Actiheart) during 9 ADL in 30 young subjects, concluded that consumer devices are not yet at the level of the best research devices: Mean bias and 95% LoA values for consumer monitors amounted to −0.55 ± 3.65 METs while for Actiheart they were 0.51 ± 2.11 METs, respectively. However, this finding is more likely confounded by the sensor location: while the Actiheart was worn on the chest, all other sensors were worn on the wrist. Indeed, when consumer and research devices are worn at the same location, differences tend to disappear. For example, a meta-analysis investigating the accuracy of wrist-worn devices found no significant overall differences between research and consumer devices in estimating EE (6). This finding is consistent with the notion that the latest generation of consumer devices incorporates similar technology to that of established research devices (41). Our findings thus contribute to the existing body of literature by demonstrating that an accelerometer integrated into a hearing aid performs comparably to a research device placed on the hip, despite exhibiting a broad LoA—a characteristic consistent with other studies in the field.

Our observation that the Audéo model marginally surpasses Fitbit and Garmin in performance should be interpreted with caution. We developed and validated our model with the assumption that 1 MET = V̇O_2_ measured individually at rest. Reanalyzing the data using 1 MET = 3.5 ml · min^−1^ · kg^−1^, mean biases for Fitbit and Garmin change from about −0.6 METs to about −0.1 METs. Precision also improves but remains slightly inferior to Audéo. More importantly, the calibration and validation groups are very similar in this study. This represents a possible disadvantage as Fitbit and Garmin were likely calibrated in a population that is not as comparable to the one used in this study. For example, EE estimation in an older population is compromised when a model is trained on younger subjects and improves substantially when the model is trained on older participants (unpublished data). This study shows that the EE estimation is slightly better in the older subgroup and that training the model on the older subgroup leads to better performance when evaluated in the older subgroup than when the model is trained on the middle-aged subgroup (data not shown). Due to age-related physiological and functional changes, e.g., changes in speed of movement, gait mechanics, and body composition (42–44), algorithms validated in younger adults may not accurately apply to older age groups (18). Whether Audéo outperforms Garmin and Fitbit (or other consumer sensors) in an independent sample in a laboratory setting or under free-living conditions, needs to be tested according to existing validation frameworks [e.g., (30)].

### 4.2 Precision of EE estimates

The broad LoA observed in this study for Audéo (and other sensors) can be attributed to the underestimation and overestimation of EE for distinct activities. Specifically, EE associated with ascending activities (e.g., climbing stairs, walking uphill) and stationary activities (e.g., cycling on an ergometer, squatting) was commonly underestimated; whereas EE for descending activities was overestimated, as illustrated in Figure 6 and Supplementary Figure S1. This phenomenon is likely attributable to the disparity between accelerometer counts and actual EE for certain activities—namely, those involving low accelerations with high EE (e.g., cycling) and those with high accelerations but low intensity (e.g., descending stairs). The variability in accuracy for single activities has also been demonstrated in reviews on the topic (6, 45) and is a known limitation when using accelerometers to predict EE. When comparing different Actigraph equations, Crouter et al. (40), for example, concluded that no single equation is valid for the EE estimation of all activities and that equations work best only in the activity subgroup they were developed. In this study, mean bias and LoA increased with activity intensity, a trend likely attributable to the nature of intense activities—predominantly ascending and stationary—which are associated with the largest errors.

In the model employed in this study, we operated under the assumption of a linear relationship between counts and METs within the different activity classes. Although the adoption of this two-step approach—initial classification followed by application of a class-specific model—is acknowledged to enhance estimation performance (46), it is not without its challenges. For example, for some activity classes, there were no or only weak correlations between counts and METs, rendering EE prediction challenging. Similarly, in a meta-analysis on the validity of the Actigraph device (worn either on the hip or wrist) for measuring EE in healthy adults, Wu et al. (45) found no correlation between activity counts and EE for some activities, e.g., during cycling, standing, walking at a moderate speed, and fast running (47). Because of the missing linear relationship between counts and METs in the wrist data within most of the activity classes in this study, we refrained from developing an EE-estimation model for ZurichMove sensors on the wrist. It is important to note that our methodology was specifically tailored to enhance the EE prediction for a sensor integrated into a hearing aid with limited memory and computational power, suggesting that different strategies might have been effective for wrist-worn sensors or devices with more computational power.

### 4.3 EE estimation over a day

Despite this variability in accuracy for single activities, the errors might cancel out under the assumption that a wide range of activities are performed over a day. Indeed, the calculated mean bias for Audéo for a 10 h wear time amounted to an overestimation of 61 kcal (∼5% of 10h-EE). Similarly, Härtel et al. (48), investigating the kmsMove sensor (worn on the hip) during rehabilitation activities over 7 h in 7 middle-aged adults, found an average underestimation of 14 kcal. Berntsen et al. (49), using Actigraph (worn on the hip), ActiReg (chest and thigh), and ikcal (chest) monitors during free-living lifestyle and working activities over 2 h in 20 younger and middle-aged adults, found mean biases ranging from −34 to −111 kcal. Despite good overall accuracy, drawing inferences for individual subjects remains challenging, as evidenced by our data (LoA 302 kcal) and Härtel's and Bernsten's findings (LoA ranging from 261 to 397 kcal). Nonetheless, it can be argued that the level of accuracy and precision shown by Audéo, and other devices is acceptable in the context of health interventions. For example, in order to achieve weight loss, energy intake should be reduced by about 500–1,000 kcal a day (50). This change is higher than the reported LoA of this study, hinting at the potential for detecting such changes. Furthermore, the observed significant within-subject correlation between an increase in intensity from walking to running, as measured by both the ergospirometric device and the Audéo sensor, underscores the device's ability to detect MET changes. However, this detection is relative rather than absolute, as indicated by the regression line's deviation from the identity line. This deviation from the identity line is the result of an overestimation of METs during level walking activities.

### 4.4 Future directions

Our findings from a sensor located at ear-level open the door for other application fields in this age range, e.g., IMU integration in in-ear headphones worn during sporting activities (29). Accuracy and precision for the Audéo sensor, and in general for accelerometers aiming to predict EE in a variety of activities (including ADLs, sporting activities), can be improved by the incorporation of heart rate (HR) data, as evidenced by O’Driscoll et al.'s (6) meta-analysis. This is particularly relevant for activities exhibiting a disparity between accelerometer counts and actual EE. It would be interesting to explore whether an ear-worn device that can detect HR via photoplethysmography can provide better performance. In addition, incorporating an altimeter has the potential to improve accuracy given that the largest errors in this study were found for ascending and descending activities. This is supported by Duncan et al. (51) who found that using barometers and global positioning systems improved EE estimation accuracy during field-based activities, compared to accelerometry alone, by 11%.

### 4.5 Limitations

This study was performed in a laboratory setting, following the framework proposed by Keadle and colleagues (30). However, it is known that the accuracy of models validated under laboratory conditions decreases when applied in free-living conditions (21). Also, the modeling approach adopted in this study did not enable the development of an EE estimation model for the ZurichMove sensors positioned on the wrists, thereby precluding a comparative analysis of their performance with the Audéo sensor.

### 4.6 Conclusion

This study demonstrates that an accelerometer integrated into a hearing aid (Audéo) can accurately estimate EE across a broad range of ADL in a middle-aged to older population. However, the precision of these estimates is limited, making personal-level inferences challenging, though still offering valuable insights on a population level. Moreover, the Audéo sensor's performance in EE prediction, using the same modeling approach, matched that of a research device worn on the hip and slightly outperformed two wrist-worn consumer monitors. This indicates that an accelerometer integrated into a hearing aid can serve as an equivalent alternative for monitoring physical activity. This opens the door to unobtrusive evaluation of energy expenditure during daily life in older individuals that are already using hearing aids, and eventually to implementation of personalized interventions promoting healthier aging.

## Data Availability

The original contributions presented in the study are included in the article/**Supplementary Material**, further inquiries can be directed to the corresponding author.

## References

[1] Caspersen CJ Powell KE Christenson GM. Physical activity, exercise, and physical fitness: definitions and distinctions for health-related research. Public Health Rep. (1985) 100:126–31. 3920711 PMC1424733

[2] Warburton DE Nicol CW Bredin SS. Health benefits of physical activity: the evidence. CMAJ. (2006) 174:801–9. 10.1503/cmaj.051351 16534088 PMC1402378

[3] Federal Statistical Office. Schweizerische Gesundheitsbefragung 2022. Bundesamt für Statistik. (2023). p. 1–28.

[4] Daskalopoulou C Stubbs B Kralj C Koukounari A Prince M Prina AM. Physical activity and healthy ageing: a systematic review and meta-analysis of longitudinal cohort studies. Ageing Res Rev. (2017) 38:6–17. 10.1016/j.arr.2017.06.003 28648951

[5] Fuller D Colwell E Low J Orychock K Tobin MA Simango B Reliability and validity of commercially available wearable devices for measuring steps, energy expenditure, and heart rate: systematic review. JMIR Mhealth Uhealth. (2020) 8:e18694. 10.2196/18694 32897239 PMC7509623

[6] O’Driscoll R Turicchi J Beaulieu K Scott S Matu J Deighton K How well do activity monitors estimate energy expenditure? A systematic review and meta-analysis of the validity of current technologies. Br J Sports Med. (2020) 54:332–40. 10.1136/bjsports-2018-099643 30194221

[7] Kraal JJ Sartor F Papini G Stut W Peek N Kemps HM Energy expenditure estimation in beta-blocker-medicated cardiac patients by combining heart rate and body movement data. Eur J Prev Cardiol. (2016) 23:1734–42. 10.1177/2047487316667786 27625154

[8] Popp WL Richner L Brogioli M Wilms B Spengler CM Curt AEP Estimation of energy expenditure in wheelchair-bound spinal cord injured individuals using inertial measurement units. Front Neurol. (2018) 9:478. 10.3389/fneur.2018.00478 30018586 PMC6037746

[9] Caron N Peyrot N Caderby T Verkindt C Dalleau G. Accelerometry-based method for assessing energy expenditure in patients with diabetes during walking. J Hum Nutr Diet. (2019) 32:531–4. 10.1111/jhn.12642 30916423

[10] Sato H Nakamura H Nishida Y Shirahata T Yogi S Akagami T Energy expenditure and physical activity in COPD by doubly labelled water method and an accelerometer. ERJ Open Res. (2021) 7:00407-2020. 10.1183/23120541.00407-2020 PMC8093485 34007842

[11] Wu S Li G Du L Chen S Zhang X He Q. The effectiveness of wearable activity trackers for increasing physical activity and reducing sedentary time in older adults: a systematic review and meta-analysis. Digit Health. (2023) 9:20552076231176705. 10.1177/20552076231176705 37252261 PMC10214103

[12] Troiano RP Berrigan D Dodd KW Mâsse LC Tilert T McDowell M. Physical activity in the United States measured by accelerometer. Med Sci Sports Exerc. (2008) 40:181–8. 10.1249/mss.0b013e31815a51b3 18091006

[13] Belcher BR Wolff-Hughes DL Dooley EE Staudenmayer J Berrigan D Eberhardt MS US Population-referenced percentiles for wrist-worn accelerometer-derived activity. Med Sci Sports Exerc. (2021) 53:2455–64. 10.1249/MSS.0000000000002726 34115727 PMC8516690

[14] Freedson PS John D. Comment on “estimating activity and sedentary behavior from an accelerometer on the hip and wrist”. Med Sci Sports Exerc. (2013) 45:962–3. 10.1249/MSS.0b013e31827f024d 23594509

[15] Huberty J Ehlers DK Kurka J Ainsworth B Buman M. Feasibility of three wearable sensors for 24 hour monitoring in middle-aged women. BMC Womens Health. (2015) 15:55. 10.1186/s12905-015-0212-3 26223521 PMC4518514

[16] Swartz AM Strath SJ Bassett DR Jr O’Brien WL King GA Ainsworth BE. Estimation of energy expenditure using CSA accelerometers at hip and wrist sites. Med Sci Sports Exerc. (2000) 32:S450–456. 10.1097/00005768-200009001-00003 10993414

[17] Rosenberger ME Haskell WL Albinali F Mota S Nawyn J Intille S. Estimating activity and sedentary behavior from an accelerometer on the hip or wrist. Med Sci Sports Exerc. (2013) 45:964–75. 10.1249/MSS.0b013e31827f0d9c 23247702 PMC3631449

[18] Migueles JH Cadenas-Sanchez C Ekelund U Delisle Nyström C Mora-Gonzalez J Löf M Accelerometer data collection and processing criteria to assess physical activity and other outcomes: a systematic review and practical considerations. Sports Med. (2017) 47:1821–45. 10.1007/s40279-017-0716-0 28303543 PMC6231536

[19] Melanson EL Jr Freedson PS. Validity of the computer science and applications, inc. (CSA) activity monitor. Med Sci Sports Exerc. (1995) 27:934–40. 10.1249/00005768-199506000-00021 7658958

[20] Bureau of Labor Statistics. American Time Use Survey. (2008). (Retrieved June 16, 2020).

[21] Guediri A Robin L Lacroix J Aubourg T Vuillerme N Mandigout S. Comparison of energy expenditure assessed using wrist- and hip-worn ActiGraph GT3X in free-living conditions in young and older adults. Front Med (Lausanne). (2021) 8:696968. 10.3389/fmed.2021.696968 34532327 PMC8438201

[22] Pasta A Szatmari TI Christensen JH Jensen KJ Pontoppidan NH Sun K Clustering users based on hearing aid use: an exploratory analysis of real-world data. Front Digit Health. (2021) 3:725130. 10.3389/fdgth.2021.725130 34713197 PMC8521852

[23] Madans JH Weeks JD Elgaddal N. Hearing difficulties among adults: United States, 2019. NCHS Data Brief, no 414. Hyattsville, MD: National Center for Health Statistics. (2021). 10.15620/cdc:107540 34319870

[24] Reed NS Garcia-Morales EE Myers C Huang AR Ehrlich JR Killeen OJ Prevalence of hearing loss and hearing aid use among US medicare beneficiaries aged 71 years and older. JAMA Netw Open. (2023) 6:e2326320. 10.1001/jamanetworkopen.2023.26320 37505496 PMC10383002

[25] Kuo P-L Di J Ferrucci L Lin FR. Analysis of hearing loss and physical activity among US adults aged 60–69 years. JAMA Network Open. (2021) 4:e215484. 10.1001/jamanetworkopen.2021.5484 33871617 PMC8056278

[26] Atallah L Leong JJ Lo B Yang GZ. Energy expenditure prediction using a miniaturized ear-worn sensor. Med Sci Sports Exerc. (2011) 43:1369–77. 10.1249/MSS.0b013e3182093014 21200349

[27] Bouarfa L Atallah L Kwasnicki RM Pettitt C Frost G Yang GZ. Predicting free-living energy expenditure using a miniaturized ear-worn sensor: an evaluation against doubly labeled water. IEEE Trans Biomed Eng. (2014) 61:566–75. 10.1109/TBME.2013.2284069 24108707

[28] Leboeuf SF Aumer ME Kraus WE Johnson JL Duscha B. Earbud-based sensor for the assessment of energy expenditure, HR, and VO2max. Med Sci Sports Exerc. (2014) 46:1046–52. 10.1249/MSS.0000000000000183 24743110 PMC3996514

[29] Choi JY Jeon S Kim H Ha J Jeon GS Lee J Health-related indicators measured using earable devices: systematic review. JMIR Mhealth Uhealth. (2022) 10:e36696. 10.2196/36696 36239201 PMC9709679

[30] Keadle SK Lyden KA Strath SJ Staudenmayer JW Freedson PS. A framework to evaluate devices that assess physical behavior. Exerc Sport Sci Rev. (2019) 47:206–14. 10.1249/JES.0000000000000206 31524786

[31] Lee PH Macfarlane DJ Lam TH Stewart SM. Validity of the international physical activity questionnaire short form (IPAQ-SF): a systematic review. Int J Behav Nutr Phys Act. (2011) 8:115. 10.1186/1479-5868-8-115 22018588 PMC3214824

[32] Weir JB. New methods for calculating metabolic rate with special reference to protein metabolism. J Physiol. (1949) 109:1–9. 10.1113/jphysiol.1949.sp004363 15394301 PMC1392602

[33] Brønd JC Anderesen LB Arvidsson D. Generating ActiGraph counts from raw acceleration recorded by an alternative monitor. Med Sci Sports Exerc. (2017) 49:2351–60. 10.1249/MSS.0000000000001344 28604558

[34] Khusainov R Azzi D Achumba IE Bersch SD. Real-time human ambulation, activity, and physiological monitoring: taxonomy of issues, techniques, applications, challenges and limitations. Sensors (Basel). (2013) 13:12852–902. 10.3390/s131012852 24072027 PMC3859040

[35] Ainsworth BE Haskell WL Herrmann SD Meckes N Bassett DR Jr Tudor-Locke C 2011 compendium of physical activities: a second update of codes and MET values. Med Sci Sports Exerc. (2011) 43:1575–81. 10.1249/MSS.0b013e31821ece12 21681120

[36] Aguilar-Farias N Peeters G Brychta RJ Chen KY Brown WJ. Comparing ActiGraph equations for estimating energy expenditure in older adults. J Sports Sci. (2019) 37:188–95. 10.1080/02640414.2018.1488437 29912666 PMC6298850

[37] Müller MJ Bosy-Westphal A Klaus S Kreymann G Lührmann PM Neuhäuser-Berthold M World health organization equations have shortcomings for predicting resting energy expenditure in persons from a modern, affluent population: generation of a new reference standard from a retrospective analysis of a German database of resting energy expenditure. Am J Clin Nutr. (2004) 80:1379–90. 10.1093/ajcn/80.5.1379 15531690

[38] Garber CE Blissmer B Deschenes MR Franklin BA Lamonte MJ Lee IM American College of sports medicine position stand. Quantity and quality of exercise for developing and maintaining cardiorespiratory, musculoskeletal, and neuromotor fitness in apparently healthy adults: guidance for prescribing exercise. Med Sci Sports Exerc. (2011) 43:1334–59. 10.1249/MSS.0b013e318213fefb 21694556

[39] Bland JM Altman DG. Statistical methods for assessing agreement between two methods of clinical measurement. Lancet. (1986) 1:307–10. 10.1016/S0140-6736(86)90837-8 2868172

[40] Crouter SE Churilla JR Bassett DR Jr. Estimating energy expenditure using accelerometers. Eur J Appl Physiol. (2006) 98:601–12. 10.1007/s00421-006-0307-5 17058102

[41] Chowdhury EA Western MJ Nightingale TE Peacock OJ Thompson D. Assessment of laboratory and daily energy expenditure estimates from consumer multi-sensor physical activity monitors. PLoS One. (2017) 12:e0171720. 10.1371/journal.pone.0171720 28234979 PMC5325221

[42] Baumgartner RN. Body composition in healthy aging. Ann N Y Acad Sci. (2000) 904:437–48. 10.1111/j.1749-6632.2000.tb06498.x 10865787

[43] Darbutas T Juodžbalienė V Skurvydas A Kriščiūnas A. Dependence of reaction time and movement speed on task complexity and age. Medicina (Kaunas). (2013) 49:18–22. 23652713

[44] Boyer KA Johnson RT Banks JJ Jewell C Hafer JF. Systematic review and meta-analysis of gait mechanics in young and older adults. Exp Gerontol. (2017) 95:63–70. 10.1016/j.exger.2017.05.005 28499954

[45] Wu WJ Yu HB Tai WH Zhang R Hao WY. Validity of Actigraph for measuring energy expenditure in healthy adults: a systematic review and meta-analysis. Sensors (Basel). (2023) 23(20):8545. 10.3390/s23208545 PMC10610851 37896640

[46] Crouter SE Clowers KG Bassett DR Jr. A novel method for using accelerometer data to predict energy expenditure. J Appl Physiol (1985). (2006) 100:1324–31. 10.1152/japplphysiol.00818.2005 16322367

[47] Kemp C Pienaar PR Henst RHP Roden LC Kolbe-Alexander TL Rae DE. Assessing the validity and reliability and determining cut-points of the Actiwatch 2 in measuring physical activity. Physiol Meas. (2020) 41:085001. 10.1088/1361-6579/aba80f 32886650

[48] Härtel S Gnam J-P Löffler S Bös K. Estimation of energy expenditure using accelerometers and activity-based energy models—validation of a new device. Eur Rev Aging Phys Act. (2011) 8:109–14. 10.1007/s11556-010-0074-5

[49] Berntsen S Hageberg R Aandstad A Mowinckel P Anderssen SA Carlsen K-H Validity of physical activity monitors in adults participating in free-living activities. Br J Sports Med. (2010) 44:657–64. 10.1136/bjsm.2008.048868 18628358

[50] American College of Sports Medicine. ACSM’s Guidelines for Exercise Testing and Prescription. Philadelphia, PA: Lippincott Williams & Wilkins (2013). 10.1249/JSR.0b013e31829a68cf 23851406

[51] Duncan GE Lester J Migotsky S Higgins L Borriello G. Measuring slope to improve energy expenditure estimates during field-based activities. Appl Physiol Nutr Metab. (2013) 38:352–6. 10.1139/apnm-2012-0223 23537030 PMC3753037

