# Supplementary material for: Energy expenditure estimation during activities of daily living in middle-aged and older adults using an accelerometer integrated into a hearing aid
Source: Front Digit Health. 2024 Jun 17;6:1400535. doi: 10.3389/fdgth.2024.1400535 (PMC11215182; doi:10.3389/fdgth.2024.1400535)
Supplement: Supplementary file 1 [file Datasheet1.docx]

Supplementary Material

**Energy Expenditure Estimation During Activities of Daily Living in Middle-aged and Older Adults Using an Accelerometer Integrated into a Hearing Aid**

**Stutz Jan^1^, Eichenberger Philipp A.^1^, Stumpf Nina^2^, Knobel Samuel E. J.^2^, Herbert Nicholas C.^2^, Hirzel Isabel^1^, Huber Sacha^1^, Oetiker Chiara^1^, Urry Emily^2^, Lambercy Olivier^3^, Spengler Christina M.*^,1,4^**

^1^ Exercise Physiology Lab, Department of Health Sciences and Technology, ETH Zurich, Zurich, Switzerland

^2^ Research & Development, Sonova AG, Stäfa, Switzerland

^3^ Rehabilitation Engineering Laboratory, Department of Health Sciences and Technology, ETH Zurich, Zurich, Switzerland

^4^ Zurich Center for Integrative Human Physiology (ZIHP), University of Zurich, Zurich, Switzerland

*** Corresponding author:** christina.spengler@hest.ethz.ch


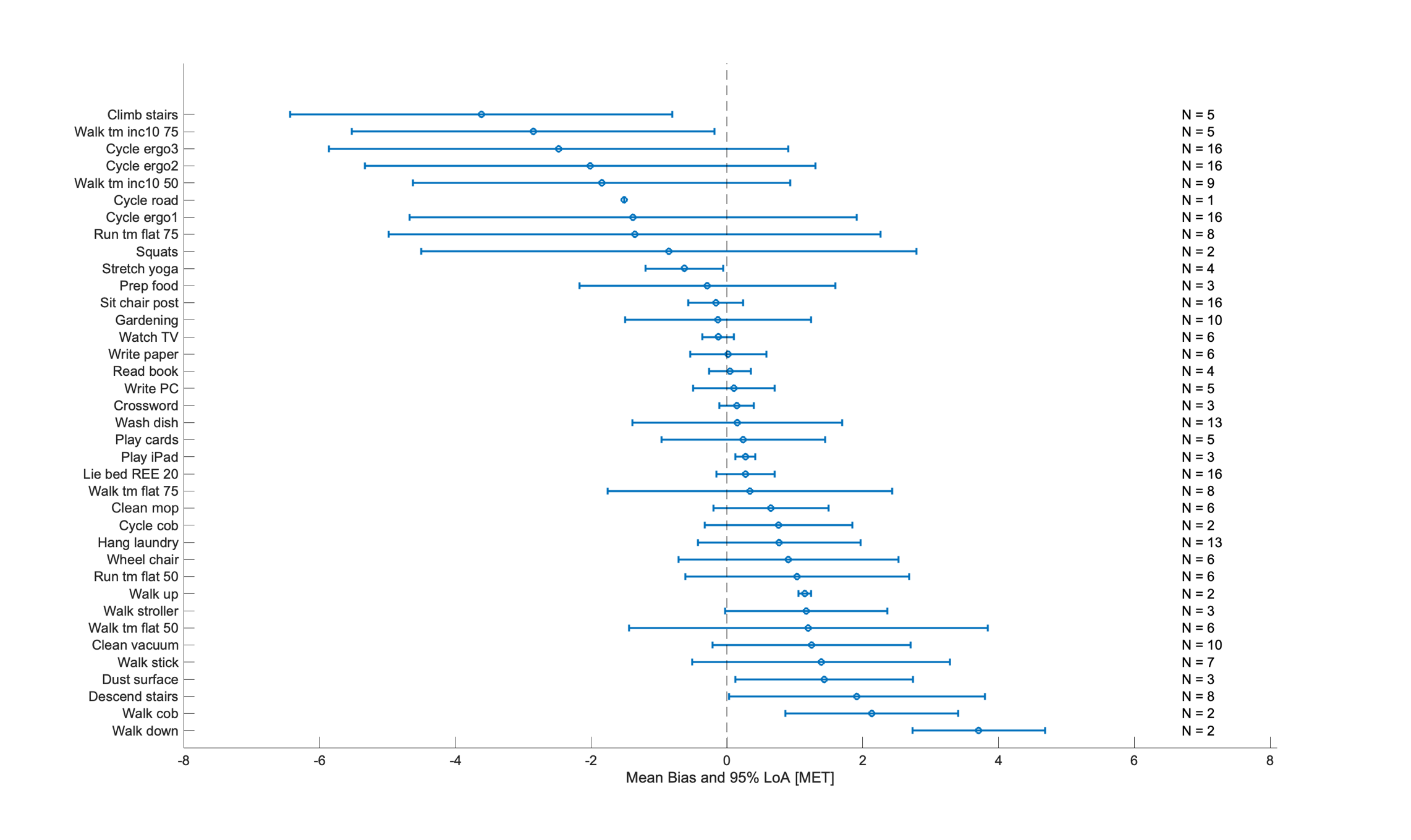


**Supplementary Figure 1.** Mean bias by activity for Audéo. *REE, resting energy expenditure; MET, metabolic equivalent of task; LoA, limits of agreement; tm, treadmill; inc10, 10% inclination, N, number of subjects (validation group)*

**Supplementary Table 1.** Participant characteristics of the calibration and validation groups

|  | Calibration group (N = 44) | Validation group (N = 16) | p-value |
| --- | --- | --- | --- |
| Age [years] | 63.9 ± 8.3 | 64.3 ± 7.5 | 0.860 |
| Sex (M/F) | 24/20 | 7/9 | 0.563 |
| Height [cm] | 171 ± 8 | 173 ± 8 | 0.623 |
| Weight [kg] | 71.8 ± 12.7 | 73.5 ± 10.9 | 0.637 |
| BMI [kg⋅m^-2^] | 24.3 ± 3.0 | 24.6 ± 2.5 | 0.724 |
| IPAQ [MET⋅min^-1^⋅week^-1^] | 2714 ± 1637 | 2804 ± 2287 | 0.867 |
| Resting V̇O_2_ [ml⋅min^-1^⋅kg^-1^] | 3.21 ± 0.64 | 3.00 ± 0.42 | 0.247 |
| Fat mass [kg] | 20.0 ± 6.5 | 22.3 ± 5.8 | 0.231 |
| Lean mass [kg] | 49.4 ± 8.9 | 48.7 ± 9.4 | 0.798 |
| BMD [T-score] | -0.06 ± 1.16 | 0.21 ± 1.04 | 0.443 |
| Systolic BP [mmHg] | 124 ± 14 | 125 ± 15 | 0.931 |
| Diastolic BP [mmHg] | 80 ± 9 | 83 ± 10 | 0.320 |
| PWV [m⋅s^-1^] | 8.7 ± 2.7 | 9.0 ± 1.8 | 0.701 |
| Handiness (R/L) | 40/4 | 13/3 | 0.370 |

Shown are means ± SD. *BMI, body mass index; V̇O_2,_ oxygen uptake at rest (activity 01, see Table 2 in main manuscript). Note that the proportion of participants tested with the Metamax 3B is comparable in both groups (11% in the calibration group and 12% in the validation group); BMD, bone mineral density, BP, blood pressure, PWV, pulse wave velocity; p-value, two-sided independent t-test for numerical variables and Fischer’s exact test for sex and handiness.*
